# A Systematic Review of Applications of Machine Learning and Other Soft Computing Techniques for the Diagnosis of Tropical Diseases

**DOI:** 10.3390/tropicalmed7120398

**Published:** 2022-11-25

**Authors:** Kingsley Attai, Yasaman Amannejad, Maryam Vahdat Pour, Okure Obot, Faith-Michael Uzoka

**Affiliations:** 1 Department of Mathematics and Computer Science, Ritman University, Ikot Ekpene 530101, Nigeria; 2 Department of Mathematics and Computing, Mount Royal University, Calgary, AB T3E 6K6, Canada; 3 Department of Computer Science, University of Uyo, Uyo 520103, Nigeria

**Keywords:** medical decision support systems, soft computing, tropical diseases, medical records, telemedicine, health science

## Abstract

This systematic literature aims to identify soft computing techniques currently utilized in diagnosing tropical febrile diseases and explore the data characteristics and features used for diagnoses, algorithm accuracy, and the limitations of current studies. The goal of this study is therefore centralized around determining the extent to which soft computing techniques have positively impacted the quality of physician care and their effectiveness in tropical disease diagnosis. The study has used PRISMA guidelines to identify paper selection and inclusion/exclusion criteria. It was determined that the highest frequency of articles utilized ensemble techniques for classification, prediction, analysis, diagnosis, etc., over single machine learning techniques, followed by neural networks. The results identified dengue fever as the most studied disease, followed by malaria and tuberculosis. It was also revealed that accuracy was the most common metric utilized to evaluate the predictive capability of a classification mode. The information presented within these studies benefits frontline healthcare workers who could depend on soft computing techniques for accurate diagnoses of tropical diseases. Although our research shows an increasing interest in using machine learning techniques for diagnosing tropical diseases, there still needs to be more studies. Hence, recommendations and directions for future research are proposed.

## 1. Introduction

There is a growing reliance on computers for decision-making in various application domains. These systems involve knowledge of an engineering process characterized by imprecision, vagueness, and approximate reasoning. This has necessitated using soft-computing techniques that model the human mind and are tolerant to uncertainty, partial truth, approximations, and imprecision to achieve robustness, reliability, traceability, and scalability [1]. Traditional computing develops exact models using symbolic logic and numerical reasoning, while soft-computing techniques use approximate reasoning and modeling [2]. The potential of soft computing techniques to identify and model meaningful relationships/patterns in a data set has made them very useful in medical diagnosis, treatment, outcome prediction, and other clinical scenarios. However, most research on the application of soft computing, and in particular, machine learning (ML) techniques, in medical diagnosis have focused on analyzing imaging results [3,4,5]. Also, more emphasis has been placed on common diseases such as diabetes [6] and cancer [7]. ML techniques are a class of soft computing techniques that enable computer programs to automatically improve their performance of some tasks through experience [8].

Medical diagnosis involves the determination of a disease or condition by analyzing the patient’s symptoms and signs [9]. Laboratory tests, radiology, biopsy, endoscopy, and others are often used to diagnose diseases. Some medical experts have also explored computerized medical diagnoses like computer tomography (CT scan). These approaches have brought about a tremendous improvement in medical diagnosis and the health domain in general. Tropical diseases reproduce rapidly in hot and humid weather and are mostly infectious diseases [10]. Tropical regions experience heavy rainfall, high temperature, and high humidity. These conditions provide a conducive ambiance for pathogenic or infectious agents to breed, affecting and influencing the living organism. Some infectious agents that cause these diseases are parasitic worms (helminths), viruses, bacteria, and protozoa. Infectious agents can be transmitted to humans through an infected human, a vector (animal or insect), or a vehicle (soil, plants, cloth, water, food, etc.) [11,12]. Tropical disease comprises communicable and non-communicable diseases, diseases caused by nutritional deficiencies or environmental conditions, and genetic disorders in these regions [13]. Common tropical diseases include malaria, diarrhea, typhoid fever, measles, lassa fever, tuberculosis, yellow fever, dengue fever, Ebola, Marburg virus, COVID-19, measles, pneumonia, hepatitis, zika virus, and influenza. The confusable nature of tropical diseases and the complications of diagnosing and managing these diseases creates a burden on frontline health workers in low-to-middle-income countries.

We aim to address this issue by developing a decision support system (DSS) based on soft computing techniques. This study allows us and other researchers with similar intentions to review the existing efforts in this domain. This study, therefore, attempts to examine the application of ML and other soft computing techniques in diagnosing tropical diseases. It is an extension of a previous study [14] that addressed five research questions relating to the application of soft-computing methods in diagnosing tropical diseases based on studies published between 2008 and 2017. Our current study covered the period 2009–2020. It also distinguishes between ML and other soft-computing techniques. It considers additional questions relating to data characteristics, sample sizes, demographic concentrations of ML systems, public availability of data, and efficiency of algorithms. The specific objectives of our study, therefore, include: (i) review the soft computing methods employed in the diagnosis of tropical diseases, (ii) determine the tropical diseases commonly diagnosed (and their features) using ML and other soft computing methods; (iii) understand the effectiveness of the algorithms, and iv) explore the limitations of the research efforts in the use of soft computing methods for tropical disease diagnosis. The paper is organized as follows: Section 2 presents the review of related literature on soft computing applied to diagnosing tropical diseases, while the research methodology is described in Section 3. The results are presented and discussed in Section 4, and conclusions are drawn in Section 5.

## 2. Related Works

In this section, we discuss related work on soft computing technologies and ML systems’ application to the diagnosis of diseases in general. We also review the literature focusing on tropical disease diagnoses.

A. Soft-computing technologies used for disease diagnosis: The first effort at developing decision support tools for medical diagnosis started with the application of statistical techniques for medical diagnosis, introduced by Lipkin, Hardy, and Engle in the 1950s [15]. By the early 1970s, the ML tools created for medical diagnosis showed evidence that statistical tools were not capable of handling complex clinical problems [16]. They laid the foundation for exploring artificial intelligence (AI) concepts in medical diagnosis. This era began with Kulikowski’s exertions in 1970 [17], which were directed at deviating from engineering approaches to intense attention of the ‘cognitive model’. Kulikowski explored the physicians’ reasoning procedures and perception in medical diagnosis [18]. Pattern recognition techniques focused on the application of AI in medical diagnosis up until Shortliffe published the first rule-based method for therapy recommendation in infectious diseases in 1974 [19]. Rule-based programs utilize the “if-then rules” in series of inferences to make conclusions. However, it was later observed that rule-based systems were only effective in facile medical domains because most critical diagnostic problems were so extensive and convoluted. Forthright attempts to link together comparatively large series of rules resulted in significant difficulties; hence, such systems were deficient of clinical reasoning [20]. As research in the application of soft computing in medical diagnosis evolved, the attention shifted to the depiction and application of imprecise, dynamic, and unstructured knowledge. The sources of information obtainable in medical DSS are characterized with imprecision and uncertainty [21,22]. These sources comprise the physician, laboratory, patient and additional technical evaluation approaches, as well as the mathematical models that mimic the diagnostic process; thus, making medical DSS researchers turn to soft computing approaches to handle imprecision and uncertainty in medical diagnosis [23]. It has been shown in [24] that AI could significantly increase frontline health workers’ diagnostic effectiveness and efficiency, especially in rural communities. A few medical decision support models have attempted to provide diagnostic advice without a physician [25,26,27]. However, these systems are largely ineffective for diagnosing tropical febrile diseases due to: (i) soft focus on tropical conditions, (ii) poor handling of confusable symptoms, (iii) unfriendly user interfaces, (iv) high reliance on internet availability, and (v) non-consideration of asymptomatic factors.
B. Tropical disease diagnosis using ML algorithms: The tropical zones of the world are more susceptible to infectious diseases than the temperate part of the world. The primary reasons why infectious diseases thrive in the tropics are due to biological and environmental influences that hold up a range of vectors, pathogens and hosts, and social drivers that weaken attempts to manage these diseases. These infectious diseases, also known as tropical diseases, are predominant in tropical regions [28]. Several tropical (especially febrile) diseases present symptoms that are very much alike, thus making these diseases “confusable.” These diseases are of immense concern to physicians, medical institutions, and the community as a whole due to the complexities of the conditions they present in early diagnosis and their mortality rates. Therefore, the use of soft computing and ML algorithms can help to prevent any misdiagnosis.

Examples of medical areas where ML techniques have been applied include:

- Care management of febrile diseases
- Finding host relationships in the cell
- Diagnosing

In the following sections, we discuss works relating to the above-mentioned categories and the differences between our work and others.

Care management of febrile diseases: Keitel et al. [29] studied the need for innovations for efficient diagnostic assessments and appropriate management of febrile children in primary care. They summarize existing Electronic clinical decision algorithms (eCDAs) to provide an overview of their validation degrees. They conclude that eCDAs are valuable tools that can improve the management of febrile disease and boost the reasonable use of diagnostics and antimicrobials. They show that the next steps in the evidence pathway should continue integrating clinically useful diagnostic and treatment innovations.

Finding host relationships in the cell: Agany et al. [30] explore the concepts of ML and data mining toward understanding vector-host pathogen relationships such as adaptation and pathogenicity. Twenty-five studies involved predictive models using supervised ML from the review articles. In contrast, 14 of the studies used unsupervised methods and deep learning. Classifying and predicting pertinent features that determine interaction outcomes were among the most dominant machine-learning tasks in the retrieved articles.

Diagnosis: The following includes a list of the commonly diagnosed diseases using soft computing techniques:

Malaria: Malaria is an acute and deadly disease attributable to a parasite that normally infects a particular type of mosquito, which feeds on humans. Malaria is a well-known cause of morbidity and mortality in tropical regions [31]. People with malaria are generally very sick with symptoms including fever, fatigue, chills, muscle pain, and shivering. Poostchi et al. [32] wrote a survey article on image analysis and ML techniques to bring up-to-date and cutting-edge developments in automated malaria diagnosis with image analysis and ML. They showed that with the advent of new deep learning approaches, the research sees a thrilling growth that can be considered revolutionary. Although considerable articles have been published in this area, Pootschi et al. believed it would render many of the former classification approaches dispensable. They also discussed that a lot of the cell segmentation techniques presented so far could soon become obsolete, and deep learning could be a promising tool. Given these developments, automated microscopy will present an easy, inexpensive, and reliable approach to diagnosing malaria.

Regarding the diagnosis of malaria fever using ML models, Oguntimilehin et al. [33] also reviewed the predictive models for the diagnosis and treatment of malaria fever. They showed that the shortage of laboratory equipment and hospitals led to many annual deaths. The study revealed that computer-based predictive models with symptoms or images of malaria parasites generated better ways to diagnose and treat malaria fever. However, most of the predictive models provide a diagnosis without therapy, and most researchers failed to evaluate the accuracy of the models. They concluded that researchers could work on symptomatic environment mobile applications so that many people could access them. Boruah et al. [34] studied the data mining applications in malaria prediction. Based on the related work, they categorized the application of ML in healthcare into Treatment Effectiveness, Healthcare Management, Fraud and Abuse, Medical Device Industry, System Biology, Hospital Management and Pharmaceutical Industry. They also classified data mining tools and techniques into Classification, Clustering, Association Rule Learning, Regression, Anomaly Detection, Summarization, Time Series Analysis, Prediction Task, and Sequence Discovery.

Dengue Fever: Dengue (or dengue fever) is a disease caused by mosquito bites as well as one of four types of dengue viruses and is a severe global health issue [35]. Dengue fever presents serious flu-like symptoms and can result in death in extreme cases. There are no vaccines against dengue fever. Therefore, soft computing poses as a better diagnostic tool. The following includes a list of literature reviews that discuss the use of soft computing and ML in disease diagnosis. Dengue fever is one of the most studied tropical diseases when applying ML models for diagnosing. Iqbal et al. [36] studied an outlook on ML for dengue outbreak prediction. They first studied all the related research work in dengue viral predication. They then proposed the development of an innovative ensemble classifier for predicting dengue fever outbreak. Sundari et al. [37] analyzed various dengue factors and reviewed research papers to identify the data mining models used to predict dengue. Considering various factors like temperature, sunshine, and rainfall, they concluded that the risk of dengue fever is linked with high temperature and is inversely related to the periods of rain and sunshine. Sivaprasad et al. [38] used the network analysis method to review articles related to early warning systems for the dengue fever outbreak. They performed a cluster analysis on the citation network using Gephi: a network analysis and visualization tool. The majority of articles fall into two clusters based on the graph: (1) the effect of climate change on mosquito-borne diseases, and (2) studies including dengue research. Ahmed et al. [39] presented a systematic review of soft computing techniques used for the identification of dengue fever and possible solutions to overcome it. The article first discussed whether expert systems correctly identify dengue fever and if knowledge-based expert systems fulfill the requirements. Finally, they discussed whether the interfaces of expert systems are user-friendly for all types of users or not. To address the mentioned goals, they concluded that although most of the works correctly diagnose the diseases, the role of knowledge-based models, which consist of two elements: diseases and their symptoms, are essential for determining disease and predicting medical suggestions related to the particular disease. Finally, they suggested that based on existing articles, user interfaces should be well-formed to convey knowledge according to the user’s mental model to have a user-friendly system.

Tuberculosis: The bacterium called mycobacterium tuberculosis causes tuberculosis [40]. The bacteria typically affect the lungs but can also affect other parts of the body, and not every infected person becomes sick. The following are the techniques used to diagnose this disease: Weiner et al. [41] have reported recent developments in the high throughput detection of tuberculosis. High-throughput methods aim to identify new biomarkers that help diagnose, treat, and prevent TB. Several studies have shown that tuberculosis manifests itself on different levels. They concluded that studies in other cohorts are needed to allow for meta-analyses and the construction of concise, universal, and predictive tuberculosis biosignatures. In another study, Doshi et al. [42] discussed how ML could transform the management of tuberculosis. ML’s integration into new software promises enabled users to benefit from artificial intelligence-enabled pattern recognition software to personalize a patient’s care plan or customize training materials. They concluded that mobile health approaches significantly impact products, and products must stay abreast of advancing technology over time.

Typhoid Fever: Typhoid fever is an infection caused by bacteria that can spread all over the patient’s body, affecting several organs and without rapid treatment, can result in serious complications and even death. Typhoid is caused by a bacterium called Salmonella typhi [43], that is related to the bacteria that cause salmonella food poisoning. Oguntimilehin et al. [44] performed a literature review on Computer-Aided Diagnostic Systems for Managing Typhoid Fever. Their study showed that typhoid fever is widespread in developing countries and is associated with many deaths. They suggested that if the developed system does not satisfy all of the factors mentioned in the paper, it may not be desirable to be used in the health sector. Finally, they suggested that the systems’ accessibility could be improved by making them web-based or mobile-based. None of the above-mentioned related works comprehensively studied all the different types of tropical diseases. This study covers most tropical diseases worldwide and the ML models used to diagnose them.

Others have done systematic reviews on tropical diseases. Akinsolu et al. [45] presented a systematic review on the emerging resistance of neglected tropical diseases (NTDs) by identifying the frequency of drug resistance for 11 major NTDs between 2000 and 2016 as well as 20 drugs for treatment within a specific period by analytically examining socio-demographic factors, resistance, and countries of relevant studies. Boyce, Katz, and Standley [46] conducted a systematic review of the Web of Science and PubMed databases to assess the risk factors for infectious diseases in the urban environments of sub-Saharan Africa. Elduma et al. [47] conducted a systematic review on dengue virus seroprevalence in Sudan and estimate the disease burden through meta-analysis. The focus of these studies is different from our work. Our study addresses questions that are not addressed in previous reviews.

## 3. Materials and Methods

In this section, we discuss the research methodology used in this study. First, Section 3.1 summarizes the steps we followed to review the literature. In Section 3.2, we discuss the study goals and our research questions. Later, Section 3.3 elaborates on our article selection strategy; Section 3.4 describes the final pool and repository of the papers we utilized in this study.

### 3.1. Overview

This systematic literature review (SLR) followed the guidelines introduced by Kitchenham and Charters [48], including the following main steps:

1. Planning the review:
  - Identifying the need for a review.
  - Specifying the research questions.
  - Developing a review protocol.
  - Evaluating the review protocol.
2. Conducting the review:
  - Identification of research.
  - Selection of primary sources.
  - Quality assessment.
  - Data extraction and monitoring.
  - Data synthesis, Meta-Analysis (MA)

**Figure 1:**
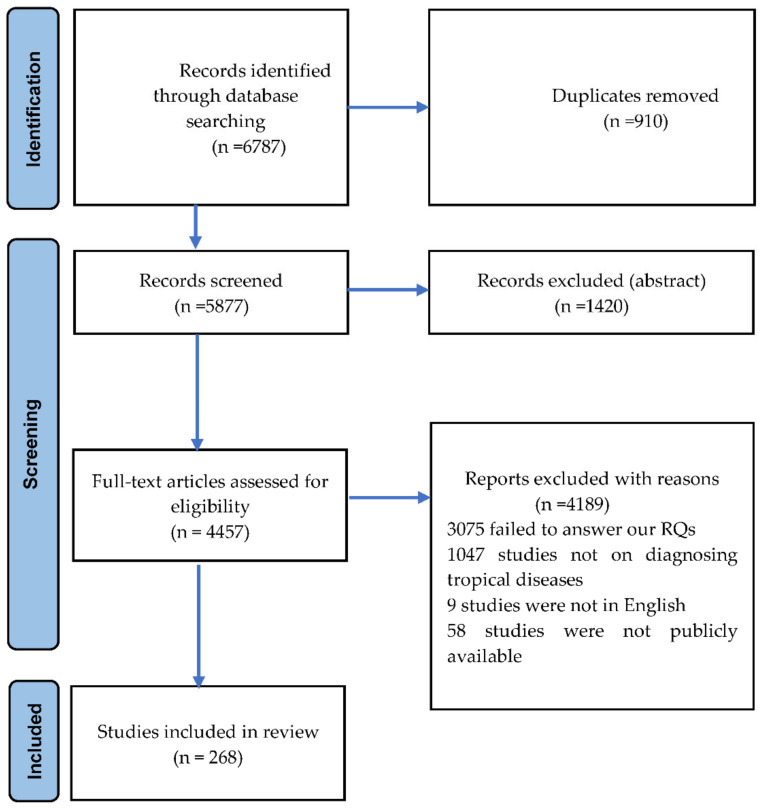
PRISMA flow chart illustrates the article search and the inclusion process.

After carefully reviewing the existing literature outlined in Section 2, we identified the gaps and the need for this review. We then specified research questions (RQs) to cover these gaps, which are explained in Section 3.2. The review process was recorded using the updated Preferred Reporting Items for Systematic Reviews and Meta-Analysis (PRISMA) guidelines [49]. The PRISMA flow chart in Figure 1 shows the systematic review’s search results and selection procedure. The PRISMA checklist is provided in the Supplementary Information. The process starts with article selection (discussed in Section 3.3). We first identify the papers based on the defined inclusion and exclusion criteria. We then finalize our paper pool and follow the data extraction and synthesis steps.

### 3.2. Goal and Research Questions

This research aims to identify the extent to which soft computing techniques such as ML models have positively impacted the quality of care that medical physicians can provide and the direct impacts on processes and outcomes related to the respective patient’s diagnosis of tropical diseases. Additionally, this research determines the causation behind the concentration and adoption of soft-computing techniques from region to region.

This research is a systematic literature review that aims to identify best practices with respect to specific procedures, technologies, methods, or tools by aggregating information from comparative studies.

The scope of our SLR study is to identify, analyze, and synthesize work published during the past ten years (from 2010 to 2020) in soft computing techniques with a focus on tropical disease diagnosis. Based on our research goal, we have formulated the following six RQs:

- RQ 1—What soft computing techniques are adopted for tropical disease diagnosis?
- RQ 2—What types of diseases are current ML systems used for?
- RQ 3—What are the characteristics of the data used for validating tropical diseases?
- RQ 3.1—What are the common sample sizes used in the studies?
- RQ 3.2—What are the current demographic concentrations for ML systems?
- RQ 3.3—What are the geographical regions covered in the studies?
- RQ 3.4—Do the validation samples contain records of both patients and non-patients?
- RQ 3.5—Is the data publicly available?
- RQ 4—What features (symptoms and characteristics) have been used for each type of disease?
- RQ 5—How efficient are the algorithms relative to the specific diseases and symptoms; how predictive are these algorithms?
- RQ 6—What are the critical limitations reported in studies related to tropical diseases?

### 3.3. Article Selection

This section briefly discusses the source article selection and search keywords used in this study and the process of applying the inclusion/exclusion criteria.

1. Source selection and search keywords: This review employs the use of the following digital and grey libraries for the search results: (1) Google Scholar 1, (2) ACM Digital Library 2, (3) PubMed 3, (4) Science Direct 4, and (5) Digital Object Identifier (DOI) Registration Agency 5. These search engines have been used in other similar studies. We used the Publish and Perish [50] tool to extract the papers. We also manually searched for the other databases (such as ACM and Science Direct) that were not supported by Publish and Perish.

The set of search terms was devised systematically and iteratively, i.e., we started with an initial set and repeatedly improved the set until no additional significant papers could be found to enhance our pool of primary studies. Considering the above aspects, we formulated our search query, as shown in Table 1. Logical operators AND/OR were used to link the search keys with the respective synonyms. The OR operator is utilized within a group, while AND is utilized amongst groups to reduce the risk of omitting relevant studies; we manually checked if we included references found in the studies within the pool.

We also extracted the names of active researchers from the initial papers found in the search engines listed above in their corresponding fields of interest. Table 2 shows the total number of available articles using the mentioned keywords.

All studies found in the additional locations that were not in the pool of selected studies but appeared to be a contender for inclusion were included in the initial pool. With the above search strings and search in specific locations, we found 268 studies, which we regarded as our initial pool of possibly relevant studies (also depicted in Figure 1). At this stage, papers in the initial collection were ready for the application of inclusion/exclusion criteria as described in the next unit.

2. Application of inclusion/exclusion criteria:

In our study, the following inclusion criteria were considered during the literature review:

- Relevance of the topic of each study to the tropical disease diagnosis concepts
- The level of comprehensiveness and evaluation followed in the study
- Whether the study was peer-reviewed

If several studies with identical titles by the same author(s) were found, the most recent study was included, and others were excluded. Only studies written in the English language within ten years (2010–2020) and those that were electronically available were included. Only the latter were included if a conference study had a more recent journal version. The relevance of each candidate study to the tropical disease diagnosis was carefully considered. All searches will be based on (1) Title, (2) Keywords, and (3) Conclusion. We also reviewed the introduction for some articles in which the data could not be inferred from the mentioned sections. Provided that these items corresponded with our criteria following analysis, the full text was obtained for further reading and data extraction.

In this study, the following exclusion criteria were considered during the literature review:

- Conference/poster abstract
- Duplicate instances of the same study
- Focus of the study does not answer RQs
- Focus is not ML for tropical disease
- Not written in English

It should also be noted that the online repository of papers (https://doi.org/10.5281/zenodo.7243308, accessed on 25 November 2022) in our pool contains a comprehensive explanation of why each article has been excluded from the primary pool (refer to the “Excluded” tab).

### 3.4. Final Pool of Articles and the Online Repository

Following the initial search and analysis for the exclusion of unrelated studies along with the inclusion of additional articles, the pool of selected articles became 268. Based on our exclusion criteria, 6787 papers were excluded, of which 1420 were conference abstracts, 910 were duplicates, 3075 studies failed to answer our RQs, 1047 of the papers’ focus were not on diagnosing tropical diseases, nine were not written in English, and 58 were not publicly available to download. The final pool of studies chosen has also been published in our online repository. Table 3 shows the number of papers in the collection by their year of publication.

## 4. Results and Discussion

A. RQ 1—What soft computing techniques are adopted to diagnose tropical diseases?

**Figure 2:**
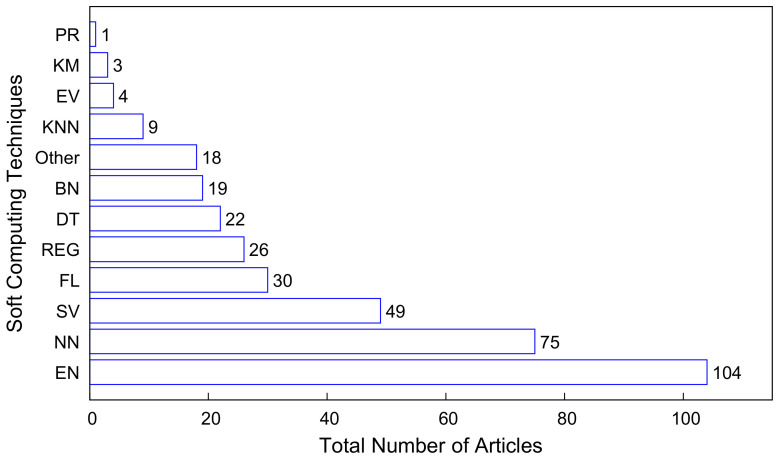
Frequencies of soft computing techniques covered in the studies.

**Figure 3:**
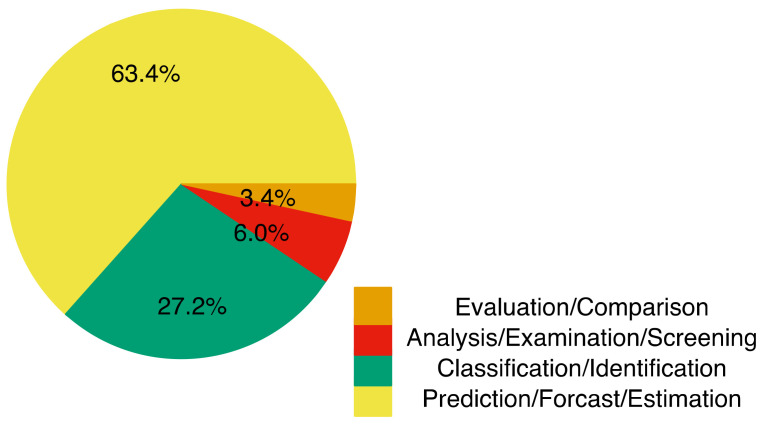
Frequency of Algorithm Goals covered by all studies.

This section summarizes the soft computing techniques covered in the studies, which were classified into 12 categories (Figure 2): Ensemble (EN), Regression (REG), Support Vector Machine and Support Vector Regression (SV), Fuzzy Logic (FL), Decision Tree (DT), Neural Network (NN), Evolutionary models (EV), Bayesian (BN), K-nearest Neighbors (KNN), K-means (KM), Probabilistic Reasoning (PR), and Other. The techniques that were categorized as ‘Other’ did not specify an exact algorithm used in the article. The various categories of techniques in the studies and their respective frequencies are illustrated in Figure 3. The highest percentage of the articles in this work used ensemble techniques for classification, prediction, analysis, diagnosis, etc. This is due to the robustness of ensemble techniques and their ability to achieve much better performance than a particular soft computing technique. The NN was the second highest due to its ability to identify hidden patterns, learn unceasingly, and enhance its capability in the process. Support Vector machine was the third highest, followed by fuzzy logic, regression techniques, decision tree, etc. The central thought about ensemble approaches is that a collection of algorithms will produce a more robust model [5]. Comparative analyses show that ensemble models outperform individual machine-learning algorithms [51,52]. Clinical investigators employ NN models in diagnosing and predicting clinical outcomes because of their suitability in modeling relationships between variables [53]. NNs have proven their potential in classification tasks, especially with the best results on various image classification tasks. SVM is best appropriate for labeled datasets and is one of the powerful algorithms widely utilized for regression and classification analysis [54]. We conducted further analysis of the soft-computing techniques in terms of the frequency of algorithms used, the goal of the study—prediction, classification, analysis or evaluation, and usage trend over the period under consideration.

Figure 3 shows the goals of the algorithms used in the study, such as prediction, classification, analysis, and evaluation as well as their respective frequencies. Of the algorithms used in the study, 63.4% handled prediction, forecasting, and prognosis (categorized into one group); this category focused on the prognosis and prediction of diseases such as malaria, typhoid, dengue fever, and other tropical diseases [55,56,57]. The second category (27.2%) grouped articles with algorithm goals like identification and classification of dengue fever and other tropical diseases [58,59,60]. The third category (6%) had the following goals: analysis of dengue fever, screening, and examining malaria, etc. [61,62]. The final 3.4% of grouped articles compared mosquito-borne disease episodes [63]. To have a logical basis for comparing machine-learning techniques with other soft-computing techniques, we grouped all the machine-learning methods into one group separate from the other soft-computing techniques.

**Figure 4:**
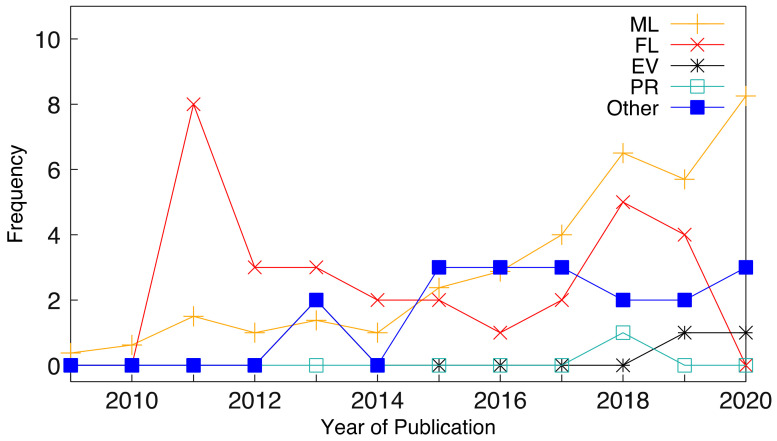
The frequency of soft computing techniques covered by all studies.

We then found the average of the frequencies for the ML techniques (ML). Other soft-computing methods include fuzzy logic (FL), evolutionary models (EV), and probabilistic reasoning (PR), and the techniques that did not fall within these categories were grouped into a class called “Other.” Figure 4 shows the trend line of the soft computing techniques (ML, FL, EV, PR, Other), the year of publication of all the studies, and their respective R_2_ values (0.8384, 0.0005, 0.0734, 0.0935, and 0.6157). The results showed that 83.8% of the ML data fit the regression model, and the trend line showed an increase in the usage of ML techniques and their application in the prediction and prognosis of tropical diseases. 0.05% of FL data fit the regression model, and those non-soft computing techniques are categorized as ‘Other’. Of the data, 61.57% fit the regression model, implying that non-soft computing techniques are also applied in the prediction and prognosis of tropical diseases. Lastly, 7.34% of EV data and 9.35% of PR data fit the regression model, indicating that these two techniques are seldom used in this research area.

B. RQ 2—What types of diseases are current ML systems used for?

To answer this research question, this section first explores disease frequency covered by all of the studies, then focuses on the frequency of the ML models used for diagnosing each disease.

**Figure 5:**
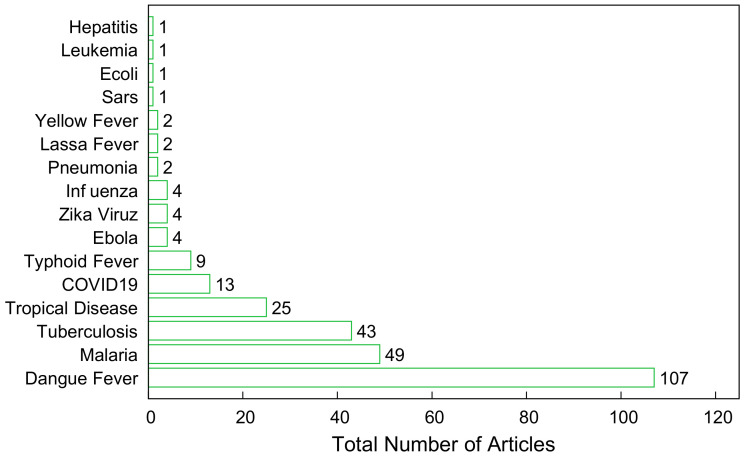
Frequency of diseases reported in the study.

Figure 5 shows the total number of diseases covered. As shown, dengue fever, with 107 studies, is the most studied disease among all tropical conditions. Malaria and tuberculosis are also the second and third most frequently studied diseases, with frequencies of 49 and 43, respectively. According to Rupali [12], malaria and tuberculosis are significant infections in the tropics, and the varying rainfall patterns and upsurge in temperatures have resulted in creating a suitable environment for vector-borne diseases, specifically, dengue and malaria.

**Figure 6:**
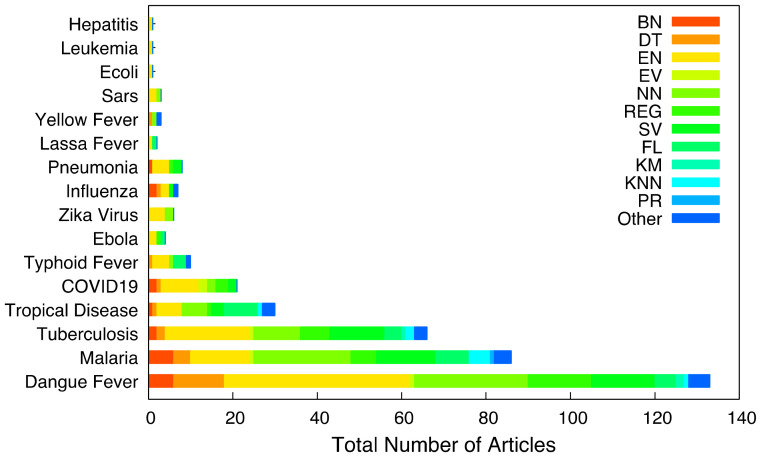
Algorithms distribution for each disease.

Figure 6 shows the distribution of algorithms used for diagnosing the studied tropical diseases such as Tropical Disease (TD), Dengue Fever (DF), Tuberculosis (TB), Typhoid Fever (TF), Yellow Fever (YF), Zika Virus (ZV), Lassa Fever (LF), etc. Some articles in the algorithm distribution for diagnosis did not specify the tropical disease diagnosed but generalized them as ‘tropical disease’, hence the tropical disease (TD) category.

**Figure 7:**
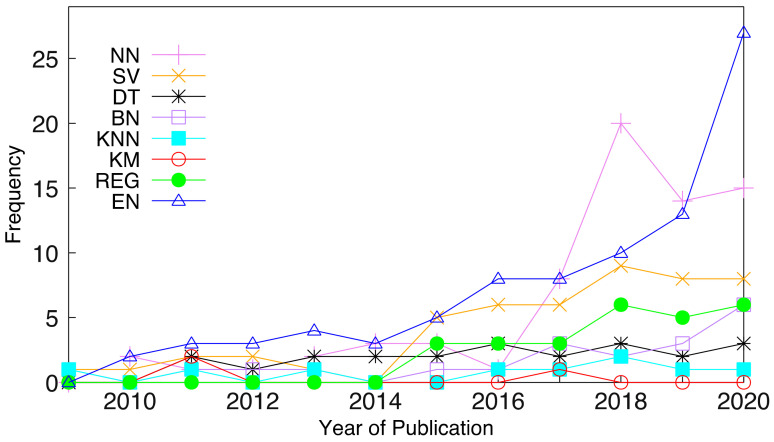
ML techniques covered by all studies.

Additionally, Figure 7 shows the trend line of the ML techniques (NN, SV, DT, BN, KNN, KM, REG, and EN). Their respective R_2_ values (0.6633, 0.7841, 0.6527, 0.5701, 0.1814, 0.0333, 0.8371, and 0.7041) show that 83.71% of the regression techniques (REG) data fit the model and the graph shows a steady rise in the use of regression techniques. Of the EN data, 70.41% fit the regression model and the trend line shows a rise in ensemble techniques due to their efficiency and robustness over other single ML techniques. Of the various data, 78.41% of the SV data, 66.33% of the NN data, 65.30% of the DT data, and 57.01% of the BN data fit the regression model. In addition, 18.14% of the KNN data and 3.33% of the KM data fit the regression model. This clearly shows the trend in the usage of ML techniques over a period of eleven years (2010–2020) and can further guide researchers on the most utilized techniques when it comes to decision-making in the medical domain.

C. RQ 3—What are the characteristics of the data used for validating tropical diseases?

We subdivided this research question into sub-questions based on the following sample characteristics: sample sizes, sample demography, geographic regions covered, control sample (patients vs. non-patients), and public availability of study data.

1. RQ 3.1—What are the common sample sizes used in the studies?

A key attribute of ML techniques is that the accuracy of results improves based on the quality and the size of the dataset. Based on the law of large numbers, the accuracy of observations improves as the number of trials increases [64]. Moreover, the accuracy of ML techniques usually improves as the sample size increases. Increasing the sample sizes or using an adequate dataset for predictive model construction can result in better prediction accuracy [65].

The predictive model constructed in [66] with a smaller dataset recorded the highest error, while the models with more datasets recorded better accuracy. The method used in [67], allocated higher weights to data points associated with larger sample sizes and the weighted methods yielded a more accurate prediction. Table 4 specifies the range of sample sizes used in all the articles of this study. Of the articles, 36 had small sample sizes of less than 101 data points. Sixty-eight articles were within the 101–1000 sample size range, 76 articles had sample sizes above 1000, and 88 of the articles did not indicate any information about the sample sizes used. The dataset size is imperative, especially in classification tasks, because some ML algorithms require small datasets while others need large datasets to provide better accuracy. Rácz, Bajusz, and Héberger [68] illustrate the effect of dataset size in multiclass classification and the findings clearly show the differences in the dataset sizes and not just in the ML techniques applied. An experiment by Wang, Fan, and Wang [69] shows a traditional ML technique performing better on small data sets while the deep learning technique performs better on large datasets.

2. RQ 3.2—What are the current demographic concentrations for ML systems?

The demographic data used in the study were age, gender, and time frame. However, 219 articles, as illustrated in Table 5, did not specify the type of demographic data used. Eighteen articles used time frame with a mean time frame of six and one-half years in their studies. Fifteen articles used age and gender with the ages mostly ranging between 15 and 75 years. Another 15 of these articles used only age and one article used only gender (female) in their prediction of dengue infection. According to [70], gender and age comparisons depict dissimilar prevalence in a number of infectious diseases and different immunological responses to infectious diseases and vaccines. Age (11%), gender (5.6%), and time frame (6.3%) were the demographic information used in our study, but 77% of the studies did not specify the demographic information used. Demographic information is important in decision-making, especially in the medical field [71,72]. Wang, Berger, and Xu [73] identify high-risk groups of patients with cancer, based on cancer types that are most vulnerable to COVID-19 based on demographic factors. An algorithm by Pourhomayoun and Shakib [74], predicted the mortality risk with demographic information, symptoms, and patients’ physiological conditions.

3. RQ 3.3—What are the geographical regions covered in the studies?

**Figure 8:**
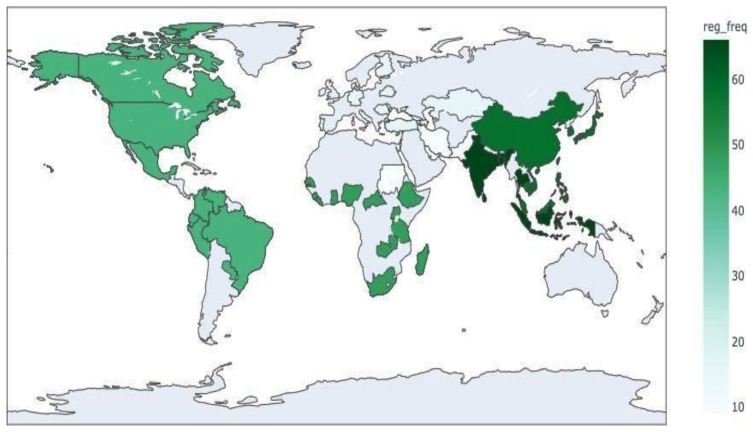
WHO Regions Heat-map.

The World Health Organization (WHO) divides the world into six regions [75], for reporting, analysis, and administration. As shown in Table 6, in this study, 49 countries were covered in all of the studies. Figure 8 shows the heat map for the frequency of studied diseases based on WHO regions [75]. In this figure, the distribution of papers is as follows: South-East Asia Region with 66 studies, Western Pacific Region with 58 studies, African Region with 48, Region of Americas with 43, European Region with 16, Eastern Mediterranean Region with 9, and 57 studies did not specify the region under study. Table 7 shows the total number of cases in the latest outbreak for each disease. Given the total number of papers that studied each disease, we can see that although malaria had a more significant number of cases in its outbreak in 2019, dengue is the most studied disease in the literature whereas pneumonia is one of the least studied yet has the highest number of cases. Several risk factors such as smoking, alcoholism, chronic medical conditions, chronic obstructed pulmonary diseases, viral infections of the respiratory tract, immunodeficiency, aging, and contact with contaminated hospital materials predispose an individual to pneumonia [76,77] and most cases of bacterial pneumonia can be treated with over-the-counter oral antibiotics [78]. This table can show the potential for studying each disease and addressing the lesser-studied diseases such as pneumonia. Additionally, the total number of articles correlates with the number of cases in recent outbreaks with a p-value of 0.006192. Therefore, the result is significant at *p* < 0.05.

4. RQ 3.4—Do the validation samples contain records of both positive and negative patients?

**Figure 9:**
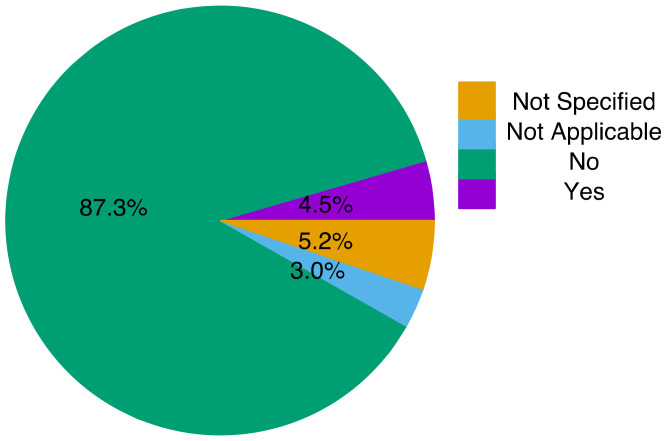
Dataset classification based on positive and negative case records.

According to the data, 14 studies did not specify any information about the positive and negative cases. Eight studies used another type of dataset, such as climate, that cannot be classified as either positive or negative. Among the remaining work, which indicate both positive and negative cases, 234 studies only used the positive cases and only 12 used both positive and negative cases. Figure 9 shows the percentage distribution of the studies. The label “YES” indicates that the studies used positive cases, and label “NO” indicates that the cases used both positive and negative cases. Labeled datasets are essential for accurate decision-making, especially for supervised learning, which requires training of the datasets. Supervised learning deduces a function from labeled training data comprising a set of examples [79] for the accurate prediction of medical conditions [80]. Therefore, using an evenhanded dataset for training and testing of a model increases the performance of an ML model [81], but in the event of an imbalanced dataset, the confusion matrix can be an effective evaluation criterion for measuring the performance of a model [82].

5. RQ 3.5—Is the data publicly available?

**Figure 10:**
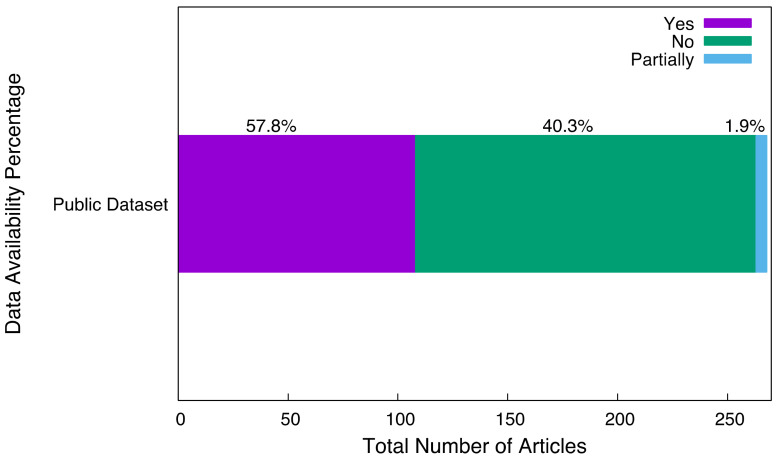
Dataset availability percentages provided by the studies.

As shown in Figure 10, among all articles, 155 papers did not make their data public, 108 papers made the data publicly available or the data was provided by a referenced paper, while 5 papers were provided as part of the data used.

D. RQ 4—What features (symptoms and characteristics) have been used for each type of disease?

**Figure 11:**
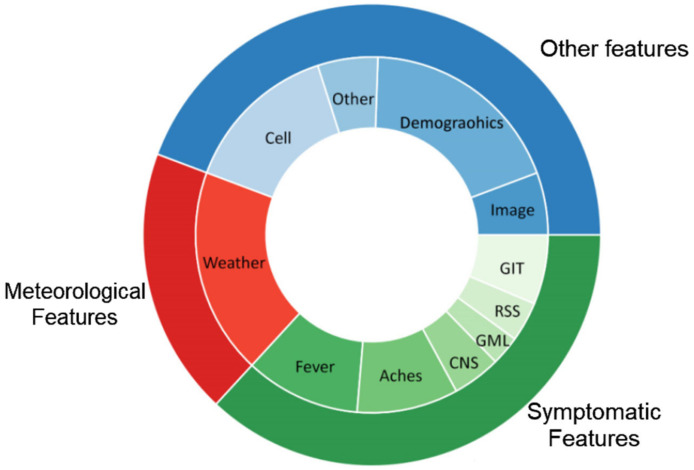
Categories of features considered in the studies.

As shown in Figure 11, the features and characteristics that were used in all the studies could be categorized into three categories: symptomatic features, meteorological features, and other features. Each study might have used more than one feature category in their study, and the statistics are based on the overall features used in each category. It is also worth mentioning that 12 studies did not specify the features used in their study. Each of these categories includes subcategories, as discussed below.

1. Symptomatic Features: 6 Symptomatic features were mainly included in the studies: Fever, Aches, Central Nervous System (CNS), Gastrointestinal Tract (GIT), Respiratory System (RSS), and General Malaise (GML). Among all of the studies, 69 included fever and 60 included ache-related features, whereas CNS, GIT, RSS and GML were included in 28, 17, 23 and 40 articles, respectively. Each subcategory is defined as follows:

- Febrile: Fever, Sweating, Shivering
- Aches: Headache, Muscle ache, Backache, Joint Pain
- CNS: Chills, Nausea, Delirium, Tiredness, Excessive Sleeping, Dizziness
- GIT: Vomiting, Diarrhea, Dehydration, Stomach Discomfort
- RSS: Abnormal Breathing, Coughing
- GML: Loss of Appetite, Yellowish Eyes, State of unwellness

2. Meteorological Features: Meteorological features mainly included humidity, rainfall, temperature, month, wind speed, altitude, and climate. Overall, 120 studies included weather data features.
3. Other Features: All other features are categorized as follows.

- Cell: Blood, Cell, URI, Hematocrit, Platelet, Protein, Gene, Genotype, Globulin, Albumin, and any other feature used in the body parts.
- Demographics: Age, Gender
- Image: Use of images of lungs as the input data and use of these image features to diagnose disease.
- Other: Any other features that could not be categorized into the mentioned categories

Among all these features included in the dataset, Table 8 shows the exact number of features used by the studies for each feature.

In other words, not all of the features have been used to diagnose disease, and some have been chosen among all features. The following are the features that are actually used for diagnosing the diseases: Fever 69, Aches 60, CNS 27, GML 17, RSS 22, GIT 40, Weather 120, Image 34, Demographics 115, Cell 88, and Other 40 in these studies. Fever, Aches, CNS, GML, RSS, GIT, Weather, and Other are the features that are used for diagnosis.

**Figure 12:**
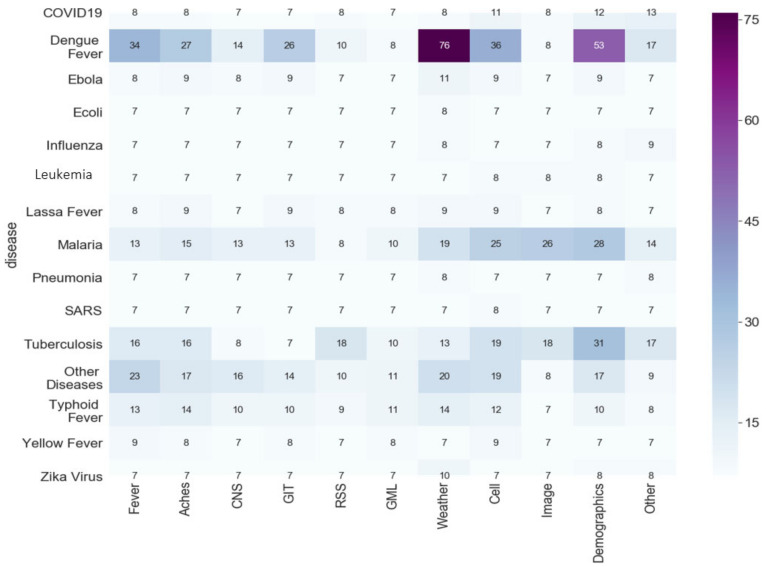
Heat Map of the diseases covered by the studies.

In order to see the correlation between the diseases and the features used in the articles, we used a heat map to show the importance of each feature per disease, as shown in Figure 12.

E. RQ 5—How efficient are the algorithms relative to the specific diseases and symptoms; how predictive are these algorithms?

**Figure 13:**
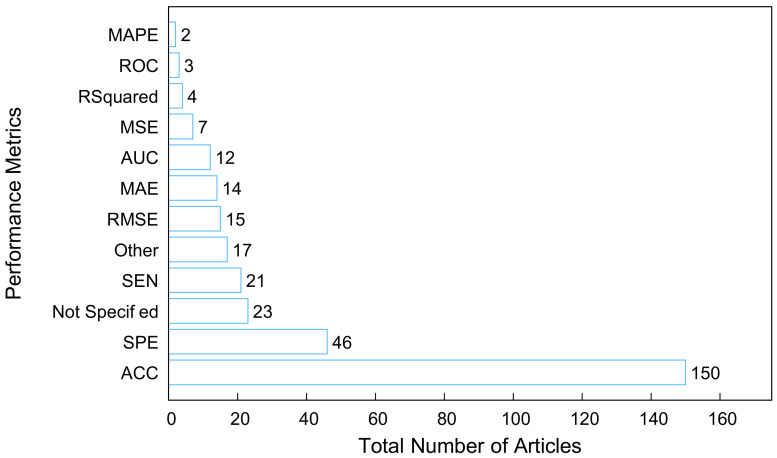
Performance Metrics provided by the studies.

Figure 13 shows the performance metrics reported. The articles categorized as “Not Specified” gave a qualitative measurement of the approach used in their study. The articles grouped as “Other” sparingly used performance metrics such as Oval, Pearson’s r, G-mean [83], window frequency [84], Akaike’s information criterion (AIC), Bayesian information criterion (BIC) [85], and Pearson correlation coefficient (PCC) in their studies.

The highest proportion of the studies used the accuracy (ACC) metric to evaluate their model. Accuracy is one of the most common metrics used to evaluate the predictive ability of a classification model. It is easy to understand, and easy to use and implement with less complexity [86,87]. Using the accuracy metric results in less optimal solutions because on the limited ability to discriminate values [88] and can also yield misleading conclusions when used with imbalanced data [89].

The second and third highest metrics used in the studies reported their performance metrics as specificity (SPE) and sensitivity (SEN) metrics. Specificity measures the probability of a negative sample being classified or the fraction of negative patterns that are correctly classified [86,90]. In comparison, sensitivity measures the probability of a positive sample being classified or the fraction of positive patterns that are correctly classified [86,90]. Sensitivity and specificity metrics can be applied in stabilizing and optimizing the accuracy performance of an imbalanced class of two class problems [90]. The sensitivity and specificity metrics could be merged into one metric (likelihood ratio) to estimate a patient’s probability of having a disease [91]. However, a positive test result could contain many false positive outcomes, which means that high sensitivity does not provide the basis for informed decisions for one to conclude that a condition is present. Conversely, high specificity does not provide the basis for making informed decisions about whether a condition is present or not [91,92].

The receiver operating characteristic curve (ROC) is a probability curve that summarizes the performance of a model, and area under the curve (AUC) signifies the degree of separability. The higher the AUC, the better the model is at prediction. AUC is a popular ranking-type metric, and its value indicates the overall ranking performance of a classifier [87].

AUC is insensitive to class distribution and may provide ambiguous results when ROC curves cross with each other [86,91].

For regression models, the mean square error (MSE) measures the difference between the predicted solutions and desired solutions. A lower MSE value indicates a better fit when evaluating a regression model. Root means square error (RMSE) is a frequently used metric in regression problems as it measures the difference between the value predicted by a model and its actual value [88]. The smaller the RMSE value, the better the performance of the model. One weakness of this metric is that a few significant errors in the sum may generate a substantial increase in RMSE, hence RMSE is not an effective indicator of average model performance and might be an ambiguous indicator of average error [93,94,95]. Table 9 summarizes the performance metrics of the algorithms relative to the techniques used in the papers under study. Note that due to the differences in the datasets used in the papers, the reported results from these papers cannot be used for identifying the outperforming methods. Such a comparison requires more in-depth analysis.

F. RQ 6—What are the critical limitations reported in studies related to tropical diseases?

In this section, we discuss the limitations of the studied articles, such as data, model, and performance limitations.

1. Stated Limitations: Among all 268 papers, only 56 stated their work limitations. The following are the three main categories discussed throughout the papers:

- Data Limitation, which includes lack of resources due to resource-poor countries as well as small samples of data and image resolution for image-based models. According to Table 4, which shows the data size range used in the study, 14% of the articles used data sizes less than 101.

As stated by [96], an ideal sample size is an imperative constituent of any research work and a study can fail to discover the existing treatment effects due to inadequate sample size. Consequently, an appropriate data size is necessary for a good result, and an unfitting data size can lead to an insignificant result. Furthermore, 33% of the study did not specify the data size used in their study, implying that almost 50% of the study used an inadequate data size and did not specify the data size used in the study. Concerning the type of dataset utilized in the study, 41% of the datasets were public, 52% did not use the public dataset, and 7% did not specify whether the dataset used was from a public or private repository. In addition, 86.5% of the study used non-patient records, 4.5% used patient records, 5.2% did not specify the type of record used, and 3.8% used weather records in their study.

- Model Limitation which includes model parameter assumption, applying a single model on the dataset, is not generalizable, and focuses on the part of data features due to the model’s nature. Among 270 papers, 13% of the articles focused on some data features, and 29% used a single model for training. Given these limitations, we may conclude that the prediction results could be more generalizable if the papers used more data features. Additionally, as many articles suggested, considering different models, and predicting the results through different models can lead us to a better result. Therefore, considering more techniques can be suggested for improving model performance.
- Performance Limitation, which includes inconsistent model replication and case-dependent results. According to the performance metrics in Figure 13, 7% of the articles were categorized as “Not Specified” because those articles did not quantify the performance metric used. 5% of the articles were categorized as “Other” because some of the studies used custom metrics and other metrics that were used did not fall under the categories of ML performance metrics listed in the study.

## 5. Conclusions

Infectious diseases severely affect tropical regions of the world. These diseases share several overlapping symptoms, thus making the diagnosis process difficult. Frontline healthcare workers working in these areas of the world can benefit from decision-support systems that can help them with disease diagnosis. Such DSSs can be developed using ML techniques. This study reviews the existing literature that uses ML techniques for diagnosing tropical diseases to highlight the efforts taken and the current research gaps.

Our study shows the increasing interest in the use of ML techniques for diagnosing tropical diseases. Dengue fever, malaria, and tuberculosis are the three diseases that are most studied in the literature. While many kinds of literature have focused on some NTDs, such as dengue fever, researchers also missed some other NTDs, such as lymphatic filariasis, schistosomiasis, trachoma, onchocerciasis, dracunculiasis, Buruli ulcer, etc. Future research studies can focus on NTDs, as well as disease outbreaks in recent years (Table 6).

ML techniques are data-driven techniques, and their performance is dependent on the availability of a good training dataset. Researchers working in this area should consider using appropriate dataset sizes to allow the model to identify and learn the patterns in the dataset since this study showcases that some of the existing literature have applied ML techniques on small datasets. To verify the effectiveness of these techniques, researchers who have access to larger datasets can apply and evaluate them in a larger setting.

## Figures and Tables

**Table 1:** Search keywords.

(“Machine learning” OR “Computer-aided” OR “Neural Network” OR “Fuzzy Logic”) AND (Tropical OR Neglected) AND (Febrile OR Fever) (“Machine learning” OR “Soft Computing” OR “Decision Tree” OR “Decision Support System”) AND (Tropical OR Neglected) AND (Febrile OR Fever) (“Machine learning”) AND (“Yellow fever” OR “Dengue fever” OR “AIDS” OR “Ebola” OR “Marburg virus” OR “Lassa fever” OR “Measles” OR “Rubella (German Measles)”) (“Machine learning”) AND (“Meningococcal infection” OR “Leptospirosis” OR “Melioidosis” OR “Escherichia coli” OR “Tuberculosis” OR “Hansen’s disease” OR “Malaria” OR “Cerebral malaria”) (“Machine learning”) AND (“Hantavirus” OR “H1N1” OR “Encephalitis” OR “Meningitis” OR “Cholera” OR “Scrub typhus” OR “Typhoid fever” OR “Rickettsia infections”) (“Machine learning”) AND (“Leishmaniasis” OR “Schistosomiasis”) (“Machine learning”) AND (“Diagnosis” OR “Consultation” OR “Assessment”) AND (Tropical OR Neglected) AND (Febrile OR Fever) AND (“Symptoms”) (“Machine learning”) AND (“Information” OR “Record” OR “Informatics”) AND (Tropical OR Neglected) AND (Febrile OR Fever) AND (“Symptoms”) (“Machine learning”) AND (“Performance” OR “Effectiveness” OR “Efficiency”) AND (Tropical OR Neglected) AND (Febrile OR Fever) AND (“Signs”)

**Table 2:** Number of articles collected from each database.

Database	Articles
Google Scholar	2130
ACM	1924
Science Direct	1600
PubMed	733
CrossRef	400
**Total**	**6787**

**Table 3:** Total Number of papers collected per year.

Year	Total of Papers
2010	6
2011	13
2012	11
2013	15
2014	9
2015	21
2016	25
2017	33
2018	46
2019	41
2020	40

**Table 4:** Range of sample sizes used in all the studies.

Sample Size	Frequency
1–30	9
31–100	27
101–1000	68
1001–5000	37
5001–10,000	14
Above 10,000	25
Not Specified	88

**Table 5:** Frequency of different demographics used in all the studies.

Demographic	Frequency (%)
Age	11
Gender	5.6
Time Frame	6.3
Not Specified	77

**Table 6:** Countries of WHO Regions that are included in all of the studied articles.

Region	Countries
African Region (AFRO)	Nigeria, South Africa, Gambia, Uganda, Tanzania, Ethiopia, Central African Republic, Zambia, Madagascar, Sierra Leone, Ghana, Senegal, Liberia.
Region of the Americas (PHOTO)	United States, Brazil, Colombia, Peru, Venezuela, Ecuador, Canada, Paraguay, Mexico.
South-East Asia Region (SEARS)	India, Indonesia, Thailand, Bangladesh, Sri Lanka
European Region (EURO)	Turkey, Portugal, Kazakhstan, Israel, Finland, Moldova, Germany, Azerbaijan, Romania, Belarus, Georgia.
Eastern Mediterranean Region (MORE)	Pakistan, Sudan, Iran
Western Pacific Region (WPRO)	China, Malaysia, Singapore, Taiwan, Vietnam, South Korea, Cambodia, Philippines, Japan

**Table 7:** Most Recent Outbreaks of Diseases and the number of papers that studied them.

Disease	Year	Total Number of Cases (Million)	Papers
Malaria	2019	229	49
Typhoid fever	2019	21	9
Dengue	2019	4.2	107
Tuberculosis	2019	10	43
HIV	2019	38	0
Leukemia	2016	60.3	1
Pneumonia	2019	150.7	2
Ebola	2016	28.616	4
COVID 19	2020	103.7	13
SARS	2003	8.096	1
Hepatitis B	2015	257	1
Hepatitis C	2015	71	0
Zika Virus	2018	1.8	4

**Table 8:** Frequency of total features and used features.

Feature	Used Features	Total Features
Fever	69	69
Aches	60	60
CNS	27	27
GML	17	17
RSS	22	22
GIT	40	40
Weather	120	120
Image	34	36
Demographics	115	119
Cell	88	91
Other	40	40

**Table 9:** Efficiency of the algorithms.

Performance Metrics	Techniques	Frequency	The Efficiency of the Algorithms
Accuracy (ACC)	BN, EN, FL, DT, NN, SV, EV, KNN, REG, Other	47.8%	85% of the studies had 75–100% accuracy, 13% of the studies had 51–74% accuracy and 2% of the studies had accuracy below 50%
Specificity (SPE)	BN, PR, NN, EN, REG, SV, KNN	14.6%	89% of the studies had specificity of 75% and above while the remaining two studies had specificity of 69% and 12%, respectively
Not Specified	FL, EN, BN, KM, NN, SV	7.3%	N/A
Sensitivity (SEN)	SV, EN, NN, KNN, BN, REG, DT, PR	6.7%	94% of the studies had sensitivity of 75% and above while the remaining 6% of the study had a sensitivity of between 69 and 72%
Root Mean Square Error (RMSE)	EN, REG, EN, SV, NN, EV	4.8%	80% of the studies had RMSE below 0.95 and the remaining 20% had MSE below 0.05
Mean Absolute Error (MAE)	EN, NN, REG	4.5%	69% of the study had a MAE value less than 20%, 23% had values between 22 and 30% and 8% had above 77%
Area Under the Curve (AUC)	REG, EN, DT, SV	3.8%	90% of the studies had AUC above 80% and the remaining 10% had an AUC of 73% and 65%
Mean Square Error (MSE)	REG, NN, EN	2.2%	75% of the studies had MSE below 0.07 and the remaining 25% had MSE below 0.82
R Squared (*R*^2^)	NN, EN, REG	1.3%	*R*^2^ values in the study where above 75% and the remaining were 10.64% and 14.9% respectively
Receiver Operating Characteristic Curve (ROC)	SV, NN, EN	1%	The study had 78%, 80% and 89% ROC values
Mean Absolute Percent- age Error (MAPE)	NN, REG	0.6%	The study had MAPE values of 0.1048 and 3.2027
Other	EN, NN	5.4%	N/A

## Data Availability

The repository of the reviewed papers and their information are listed under Supplementary Material.

## Supplementary Materials

The following supporting information can be downloaded at: https://www.mdpi.com/article/10.3390/tropicalmed7120398/s1. The papers reviewed in this literature review and the extracted data are publicly available at: https://doi.org/10.5281/zenodo.7243308. The PRISMA checklist is available at: https://doi.org/10.5281/zenodo.7262418.
